# Probing the Atomic Arrangement of Subsurface Dopants in a Silicon Quantum Device Platform

**DOI:** 10.1021/acsami.2c23011

**Published:** 2023-04-28

**Authors:** Håkon I. Røst, Ezequiel Tosi, Frode S. Strand, Anna Cecilie Åsland, Paolo Lacovig, Silvano Lizzit, Justin W. Wells

**Affiliations:** † Center for Quantum Spintronics, Department of Physics, Norwegian University of Science and Technology (NTNU), NO-7491 Trondheim, Norway; ‡ Department of Physics and Technology, University of Bergen (UiB), Allégaten 55, 5007 Bergen, Norway; ¶ Elettra-Sincrotrone Trieste, s.s. 14-km.163,5 in Area Science Park, Basovizza, Trieste 34149, Italy; § Instituto de Ciencia de Materiales de Madrid (ICMM-CSIC) C/Sor Juana Inés de la Cruz 3, 28049 Madrid, Spain; ∥ Department of Physics and Centre for Materials Science and Nanotechnology, University of Oslo (UiO), Oslo 0318, Norway

**Keywords:** delta-layers, quantum electronic devices, quantum computing, photoemission, photoelectron diffraction

## Abstract

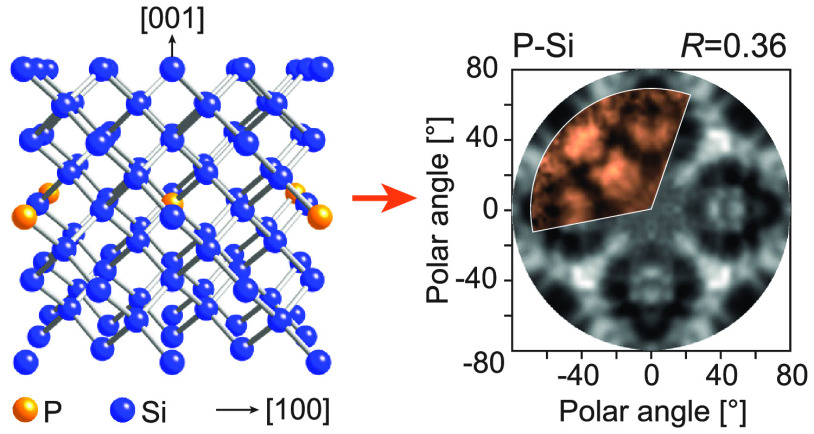

High-density structures of subsurface phosphorus dopants in silicon continue to garner interest as a silicon-based quantum computer platform; however, a much-needed confirmation of their dopant arrangement has been lacking. In this work, we take advantage of the chemical specificity of X-ray photoelectron diffraction to obtain the precise structural configuration of P dopants in subsurface Si:P δ-layers. The growth of δ-layer systems with different levels of doping is carefully studied and verified using X-ray photoelectron spectroscopy and low-energy electron diffraction. Subsequent diffraction measurements reveal that in all cases, the subsurface dopants primarily substitute with Si atoms from the host material. Furthermore, no signs of carrier-inhibiting P–P dimerization can be observed. Our observations not only settle a nearly decade-long debate about the dopant arrangement but also demonstrate how X-ray photoelectron diffraction is surprisingly well suited for studying subsurface dopant structure. This work thus provides valuable input for an updated understanding of the behavior of Si:P δ-layers and the modeling of their derived quantum devices.

## Introduction

Over the past decade, the effort to realize a silicon-based, complementary metal oxide semiconductor (CMOS)-compatible quantum computer has been intensifying,^1−3^ and several significant breakthroughs have been achieved.^4−6^ One common factor in this development is the so-called Si:P δ-layer platform,^7,8^ i.e., an ultrasharp and narrow layer of phosphorus dopants placed beneath the silicon surface, which can be patterned with atomic precision.^9,10^ The δ-layer platform can be used for quantum dots and tunnel barriers,^11^ metallic interconnects,^12^ and other key components required for quantum device engineering.^1^ This, in turn, has required it to be thoroughly studied and understood.^13−26^ Despite these intense efforts and the great progress which has been made, one key question has remained unanswered: *What is the arrangement of the dopants within the δ-layer?* The answer is of central importance for the performance of δ-layer-derived devices because the dopant arrangement is understood to directly impact key electronic properties: for example, the energy separation (i.e., “valley-splitting”) of the supported quantum well states.^27−29^

There may be multiple reasons why the atomic arrangement is not known, but we conjecture that it is primarily because, until now, a suitable probing method had not been identified. Traditional X-ray diffraction methods are unsuitable because of the atomically thin nature of the δ-layer.^30^ High-angle annular dark-field imaging with an electron microscope is also exceptionally challenging because of the similarity in atomic weight of Si and P.^31^ Recent studies have shown that the quantum confinement of the δ-layer can be ascertained by means of ellipsometry,^32^ but the in-plane coordination of the dopant atoms has remained elusive.

In this work, we demonstrate that the neighborhood around the dopants can be directly probed using X-ray photoelectron diffraction (XPD), in which a chemically specific diffractive image is formed by utilizing subtle core-level energy shifts that are concomitant with the coordination of a dopant.^33,34^ Although XPD is primarily used as a probe of surface structure,^35−37^ we demonstrate here that it also has great potential for determining the local arrangement of subsurface atoms and, therefore, is perfectly suited for solving the long-standing mystery of the Si:P δ-layer structure.

## Results and Discussion

The growth of δ-layers has been studied and refined over the recent years, not least of all to maximize the density of P atoms within the dopant plane.^23,38,39^ The basic preparation approach involves exposing a clean Si(001) surface to saturation coverage of PH_3_ gas, followed by subsequent dissociation of the gas and incorporation of P into the Si surface.^40−43^ Refinements of the method involving multiple cycles of PH_3_ exposure and P incorporation have been shown to maximize the doping density, while retaining a sharp confinement of the δ-layer.^25,39^ In all cases, the doped surface is then overgrown with undoped silicon to encapsulate the dopant layer.^13,23^

XPD, like other photoemission-based methods, is especially challenging to perform on buried atomic species because their resulting photoemission signal will be strongly attenuated by the overlayers.^44^ The attenuation problem has already been addressed specifically for Si:P δ-layers,^19,45,46^ and, although rare, XPD studies of subsurface atomic arrangements exist and have demonstrated their feasibility.^47,48^ In order to show that XPD of Si:P δ-layer structures is even possible, we therefore first focus on a δ-layer with a maximized dopant density (i.e., “double-dosed”) and with a minimized encapsulation layer thickness (i.e., ≈1 nm).

Quantitative X-ray photoelectron spectroscopy (XPS) analysis (see the Experimental Section) of the double-dosed system before, during, and after Si encapsulation reveals that a 0.39 monolayer (ML) P coverage is achieved, i.e., similar to the 0.53 ML reported previously.^39^ The same analysis also reveals that ≈90% of the P dopants remain in the δ-layer after the Si overlayer growth and final annealing steps have been completed (and the additional ≈10% segregates to the surface). From our preparation, we achieved an effective electron carrier density of *n* = 2.3 × 10^14^ cm^–2^ (see the Supporting Information for details^49^), in line with the best-case carrier density of *n* = 3.6 × 10^14^ cm^–2^ for single-layer Si:P structures.^39^

**Figure 1:**
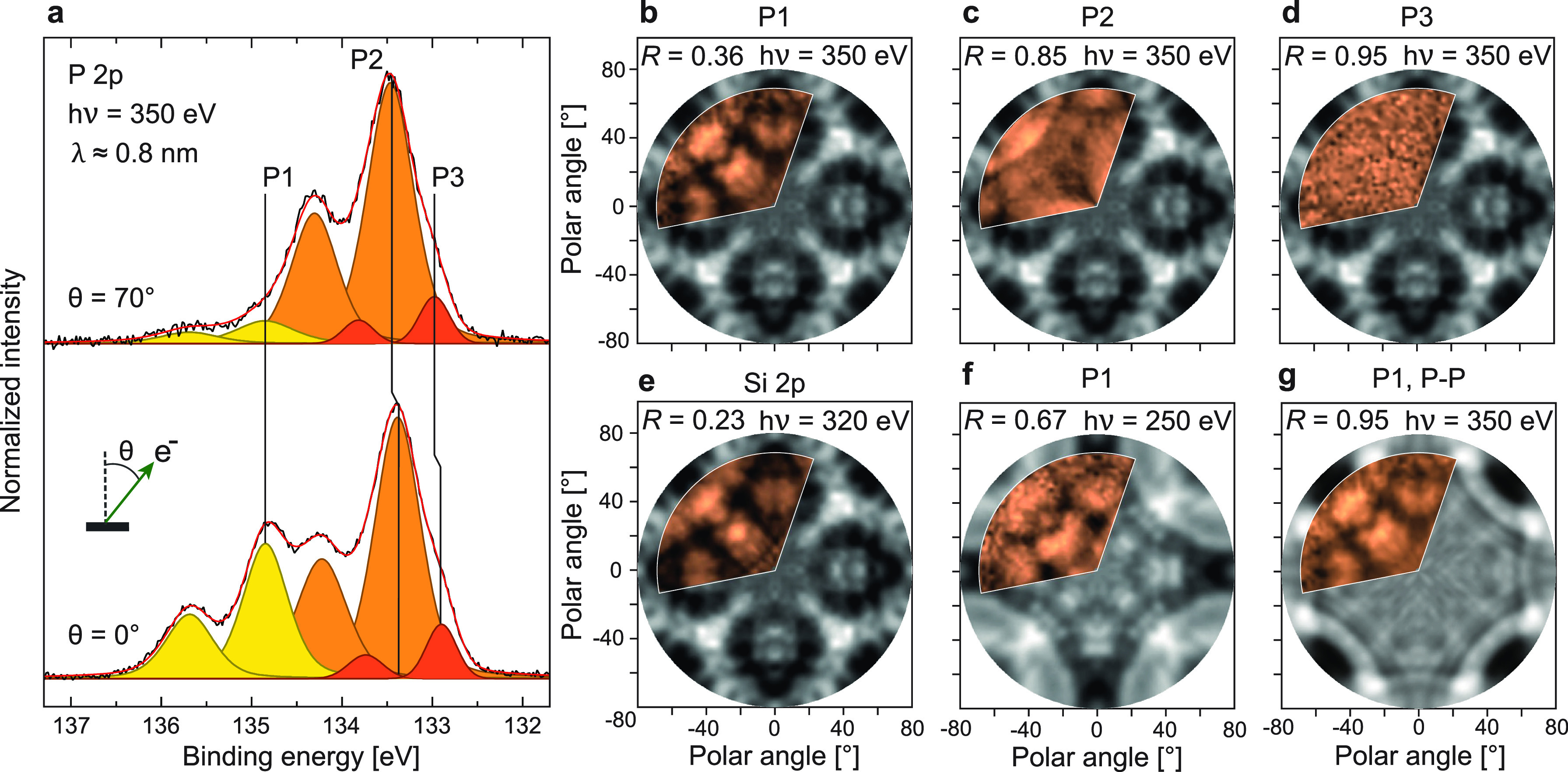
Angle-dependent photoelectron spectroscopy from a “double-dosed”, Si-encapsulated δ-layer. (a) XPS of the P 2p peak, measured with *hν* = 350 eV at normal (θ = 0°) and grazing (θ = 70°) emission and an integrated half-angle acceptance of ≤5°. P1 comes from P in the δ-layer, and P2 and P3 from P near the Si surface. Both spectra have been scaled to the intensity of P2. (b–d) The measured (orange) and calculated (gray) XPD patterns for the peaks P1–P3 from the double-dosed δ-layer system shown in (a). (e) The measured and calculated XPD from the corresponding Si 2p core level. (f) The measured XPD from P1 at *hν* = 250 eV, compared with XPD simulations of P–Si bonding (i.e., substitutional doping) within the δ-layer. (g) The measured XPD of P1 at *hν* = 350 eV from (b), compared with XPD simulations of P–P bonding (i.e., dimerization) within the δ-layer.

The XPS signal from the phosphorus 2p core level, after the completion of all of the growth steps, is shown as Figure 1a at two different photoemission angles (θ). The P 2p signal consists of three doublet components, each described by two Voigt functions with a spin–orbit splitting energy of 0.84 eV and an intensity ratio of p_3/2_:p_1/2_ = 2:1. The doublet labeled P1 at the largest binding energy (134.85 eV) represents the dopants in the buried Si:P δ-layer, whereas P2 (133.38 eV) and P3 (132.90 eV) correspond to surface phosphorus in two distinct coordinations.^49^ Although ≈90% of P is present in the buried layer, the strong attenuation of the photoemission signal from the buried dopants makes P1 look very weak in comparison with the unattenuated signals (P2, P3) from trace amounts of residual surface P.

To confirm the assignment of phosphorus components from the buried δ-layer and on the surface, the finite mean-free path (λ) of photoelectrons was exploited.^44,50^ The intensity of P1 relative to P2 and P3 appeared strongest at normal emission (θ = 0°) but was drastically reduced at θ = 70°. Assuming an intensity model *I*(*d*, θ) ∝exp{− *d*/(λ cos θ)}, the signals from dopants at a depth *d* beneath the surface should attenuate more rapidly with increasing θ when compared to the surface species. By this argument, P1 was located furthest away from the surface. Investigations as a function of the photoelectron kinetic energy led to the same conclusion.^49^

To determine their atomic arrangements both on and beneath the Si surface, XPD experiments of the P1–P3 components were performed. For this purpose, XPS measurements of P 2p were acquired over a large range of azimuthal (φ) and polar (θ) angles. Polar plots of the intensity modulation function χ were then produced alongside diffraction simulations for different dopant coordinations (details in the Experimental Section and the Supporting Information(49)).

Since the bulk structure of Si is known,^51^ XPD patterns of bulk Si 2p were also measured from the same sample and compared to their corresponding XPD simulations as a confirmation of the methodology.^49^ A bulk-sensitive Si 2p XPD pattern is shown in Figure 1e (orange) overlaid on the simulated XPD pattern (gray). Both exhibit an apparent and similar fourfold symmetry. A “reliability” factor *R* = 0.23 indicates a satisfying agreement between the two, i.e., confirming that the expected Si structure is well reproduced by our XPD simulations (see the Experimental Section for a description of the simulation optimization and a definition of the *R*-factor).

From our high-density, double-dosed δ-layer system, three XPD patterns of P 2p were obtained, i.e., one for each of the components P1–P3 (Figures 1b–d). Notably, the measured XPD pattern of P1 (Figure 1b) appeared strikingly similar to the measured XPD pattern of Si 2p at the same photoelectron kinetic energy (Figure 1e). Matching XPD patterns from the two core levels can be expected if P and Si assume similar atomic positions:, i.e, if the P1 dopant atoms replace Si atoms in the host unit cell by substitutional doping.^27,29^ The visual agreement is supported by *R* = 0.36 when comparing the measured P1 XPD with an XPD simulation of bulk substitutional doping (Figure 1b). This corresponds to an uncertainty in the P atomic positions of less than 0.1 Å.^49^

Contrary to the situation with P1, the XPD patterns from the surface components P2 and P3 are not expected to be well reproduced by this simulation. The measured patterns of P2 and P3 are shown in Figures 1c and d, respectively, overlaid on the simulated XPD from Figure 1b. Here, P2 exhibits a modulation in intensity and apparent fourfold symmetry but is otherwise in poor agreement with the substitutional doping model (*R* = 0.85). Furthermore, P3 shows almost no structure at all, as evidenced by *R* = 0.96. Hence, the achieved *R*-factors confirm that neither P2 nor P3 originated from bulk-substituted, subsurface P dopants.

The XPD patterns presented so far were obtained from photoelectrons with kinetic energies *E*_K_ ≈ 220 eV. Photoexcitation at higher energies will typically promote forward scattering along the sample surface normal and will also enhance the sensitivity to the bulk structure.^35,52^ We also performed measurements of both Si 2p and P 2p photoelectrons with lower kinetic energies (*E*_K_ ≈ 120 eV), i.e., intending to enhance backscattering of photoelectrons and a sensitivity to the surface structure.^37,53^ To no surprise, the XPD pattern was very different, and therefore the XPD simulations were further optimized to account for the apparent surface symmetry observed by surface diffraction (Figure 3d, Experimental Section). A better agreement was achieved by means of a crude surface dimer model, where the Si atoms in the topmost atomic layer were perturbed toward a partial 2 × 1 surface reconstruction,^54,55^ i.e., more consistent with the observed diffraction pattern. For the bulk Si 2p component measured at *E*_K_ ≈ 120 eV, a surface perturbation of Δ*a* = 0.3 Å gave an optimal match between the measured and the simulated XPD patterns.^49^

Comparing the P1 XPD measured at *E*_K_ ≈ 120 eV with a simulation of a substitutionally doped Si:P δ-layer having the same Δ*a* imposed at the Si surface (Figure 1f), a moderate reliability (*R* = 0.67) was achieved. The higher *R*-factor found for P1 at this kinetic energy is likely related to the reduced photoemission signal from—and hence the worse statistics for—the subsurface dopants when measured with a shallower λ. Nonetheless, the weak reconstruction provided by the simple surface dimer model led to a reasonable first approximation, where the intensity modulation and symmetry of the XPD pattern from the more surface-sensitive measurements were preserved.

In a simple model for PH_3_ dissociation on Si(001), one-in-four Si sites become occupied by a P atom, and three neighboring sites are initially occupied by H.^56,57^ This leads to the presumption that an ideal, “single-dosed” Si:P δ-layer contains 25% P. The local arrangement of P atoms within the δ-layer has been an open debate, and multiple models have been proposed.^27,29^ Several of the possible arrangements include P atoms as nearest neighbors, thus leading to the suggestion of P–P dimers, clusters, or chains.^29^ When the density of P atoms on a Si(001) surface is increased, P–P neighbors are expected to become increasingly common.^56^ This can potentially be problematic for Si:P-derived devices since P–P dimerization has been described as leading to a reduction in the overall active carrier density within a δ-layer.^39,58^

Our XPD study of encapsulated δ-layers can offer two different insights into this matter: (i) We can simulate possible structures with P–P nearest neighbors (specifically dimers and clusters) and see if this leads to an improvement in the agreement with the experimental data, and (ii) we can grow a series of samples in which the dopant density within the Si is varied.

First, the measured, bulk-sensitive XPD of P1 at *E*_K_ ≈ 220 eV was evaluated against an optimized δ-layer simulation with in-plane P–P dimers of bond length 2.42 Å (Figure 1g).^49^ Both the large reliability factor *R* = 0.95 and a visual comparison of the two patterns suggested that the measured and simulated XPD results were poorly correlated. When measured at lower kinetic energy (*E*_K_ ≈ 120 eV) where the sensitivity to the local atomic bonding is even greater,^59^ the reliability was even worse.^49^ We, therefore, infer that P–P dimerization did not occur in the δ-layer. Similarly, no convincing improvement was made using a cluster model.^49^ Hence, we conclude that nothing other than individual P atoms substituting in Si sites is needed to satisfactorily explain the experimental results.

Second, we prepared δ-layer samples using a range of recipes in order to modify the dopant density. In addition to the sharply confined, double-dosed, high-density recipe described above, we also prepared a lower P concentration single-dosed/single-layer sample^24^ with electron carrier density *n* = 5.1 × 10^13^ cm^–2^ and a multilayer sample with eight cycles of δ-layer growth and subsequent Si encapsulation (its *n* similar to that of the atomically thin δ-layers^24,25,38,58^).

**Figure 2:**
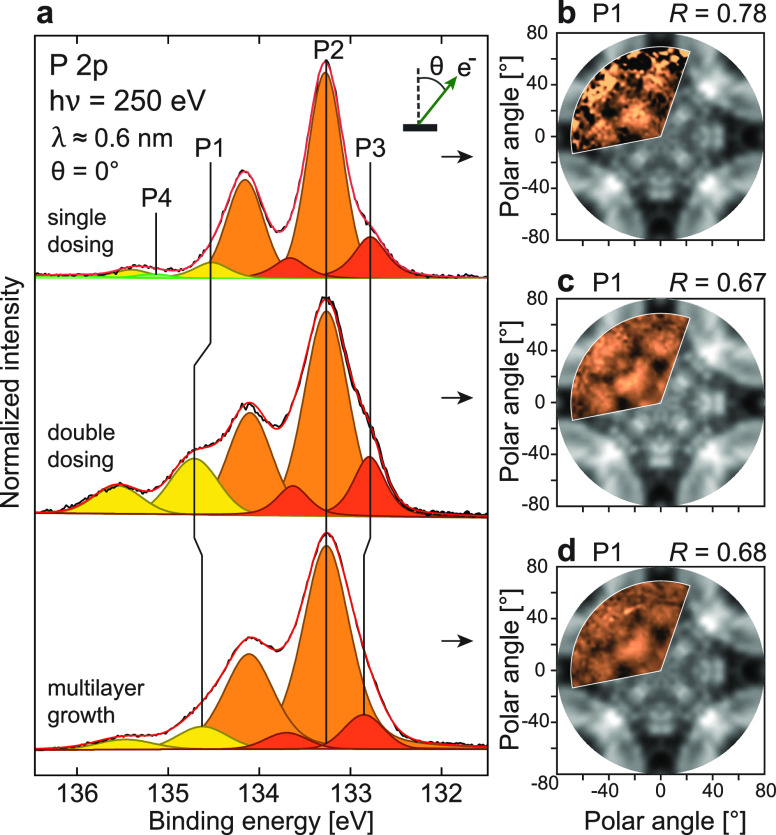
A comparison of the three different sample preparations. (a) Normal emission XPS spectra of the P 2p core level for “single-dosed”, “double-dosed”, and “multilayer” samples (top to bottom, respectively). The spectral intensities have been normalized to the P2 peak. (b–d) Corresponding, measured (orange), and simulated (gray) XPD patterns for the single-dosed (b), double-dosed, (c) and multilayer (d) samples with *hν* = 250 eV.

A comparison of the XPS and XPD structure from the three different preparations is shown in Figure 2. The P 2p core levels collected from the three samples (single-dosed, double-dosed, and multilayer) are shown in Figure 2a. Correspondingly, their derived XPD image plots are shown in Figures 2b–d. All three of the XPD patterns shown were acquired at *hν* = 250 eV and display the modulations of the P1 component from the buried dopant plane(s).

At first glance, the three XPD patterns share the same main features and symmetry as the measured and simulated Si structure.^49^ They generally have higher *R*-factors due to the reduced bulk sensitivity at this kinetic energy (*R* = 0.78, 0.67, and 0.68 for single-, double-, and multilayer dosing, respectively). Their main difference can be seen from the varying signal-to-noise ratio of each plot, originating from the different P1 component intensities. Especially the weakly doped, single-dosed sample yielded a significantly weaker signal than the other two. Given its ≈1 nm Si overlayer, the fact that its P1 shows a clear modulation at all is quite impressive. The P1 component of the double-dosed sample is noticeably stronger, conceivably from having more P dopants incorporated in its δ-layer.^39^ It also appears stronger than the multilayer P1 signal, despite there being a larger number of P dopants present in the latter system.^58^ This may be due to small differences in the overlayer thickness and the fact that all three P 2p core levels shown in Figure 2a have been normalized to their surface P2 intensities. The multilayer preparation, with its nine cycles of annealing, should lead to an increased diffusion of P atoms and the formation of more surface P.^13,58^ Resultingly, the subsurface P signal would appear relatively smaller after normalization.

The reduced bulk sensitivity at lower kinetic energies and, resultingly, the moderate *R*-factors for these three δ-layer systems well illustrate the challenge of accurately capturing all details of the doping within subsurface layers.^47,48^ We also note that our XPD simulations, while detailed, are not expected to have fully captured all details of the photoelectron diffraction process.^60^ Nevertheless, the apparent similarities between the XPD of Si and the buried P atoms measured with bulk- and surface-sensitive photon energies indicate that all three δ-layer systems exhibited a similar substitutional P incorporation and, furthermore, that no evidence of dimerization or clustering was observed.

## Conclusions

To summarize, we have first of all demonstrated how it is possible to use XPD to study the local structure around *n*-type dopants located beneath the surface of a semiconducting host. Although the dopant layer is described as “high density”, it is very narrow and contains a relatively small number of dopants (for example, ≈25% of an atomic layer). This makes it very challenging to study the structure with other methods. Having demonstrated the applicability of XPD, we have revealed that the dopants can be accurately described as P atoms substituted into Si sites within the bulk Si crystal. This is contrary to the previous postulations of in-plane P–P dimerization.^29,39^ Furthermore, we have used a range of sample preparation methods to create low-density, high-density, and multilayer dopant planes. We have shown that, in all cases, the best agreement is found by pure substitution of Si with P. Furthermore, we found no evidence to support the notion that dimerization is encouraged by increasing the dopant density or absolute dopant number.

These findings are especially important for the silicon quantum device community where Si:P δ-layers are utilized as a platform. Until now the dopant structure has not been resolved, and calculations have shown that dopant ordering (such as dimerization) is a key factor in dictating the valley splitting of the favorable quantum well states.^28,29^ We thus also conclude that XPD is a surprising, yet essential, tool for furthering the development and optimization of the much-prized δ-layer platform—^12,61−63^ and quite possibly other quantum device architectures with subsurface dopant assemblies.

## Experimental Section

### Sample Growth

**Figure 3:**
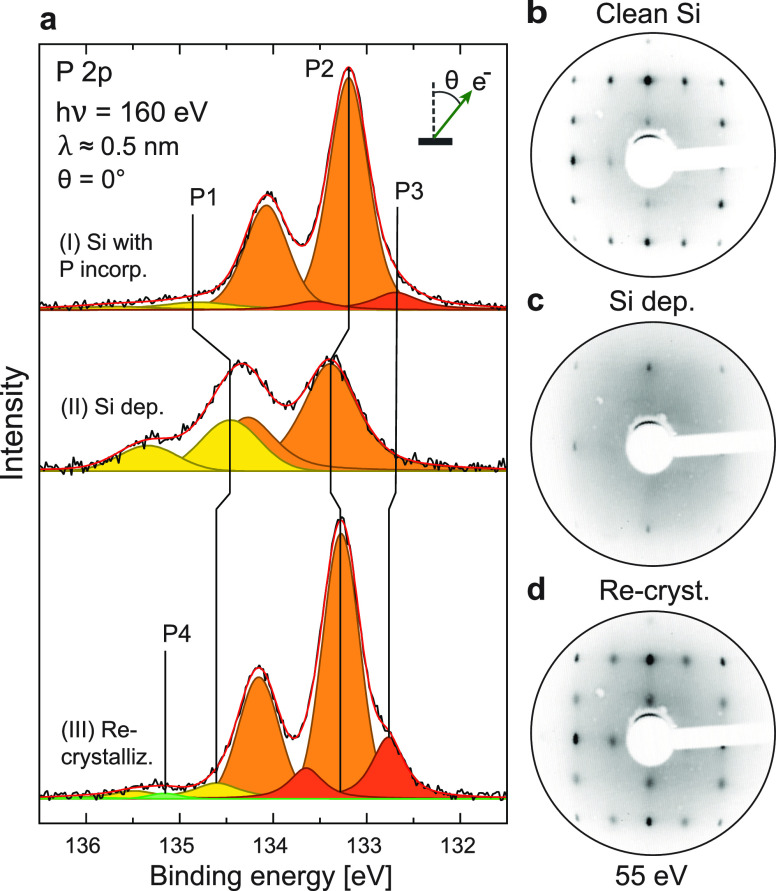
Epitaxial growth of a single-dosed Si:P δ-layer. (a) The development of the P 2p core level measured at θ = 0° and *hν* = 160 eV, upon (I) PH_3_ decomposition and dopant incorporation, (II) encapsulation/Si overlayer deposition, and (III) recrystallization of the Si overlayer. (b–d) Surface diffraction (LEED) patterns of the single-dosed δ-layer system (b) before doping, (c) after doping and Si encapsulation, and (d) after the recrystallization of the Si overlayer.

Surfaces of *n*-type Si(001) with negligible surface oxide on them were prepared in-vacuum by short cycles of high-temperature annealing to 1200 °C (measured with a pyrometer, ϵ = 0.79). The clean surfaces revealed a (2 × 1) reconstruction when investigated using low-energy electron diffraction (LEED), as shown in Figure 3b. Next, the surfaces were exposed to 1.125 Langmuirs (L) of gaseous PH_3_ (added chamber pressure of 5 × 10^–9^ mbar for 5 min) at room temperature and subsequently annealed to 550 °C to dissociate the PH_3_ and incorporate P into the Si surface.^38,42^ For the double-dosed samples, dosing of PH_3_ and subsequent annealing to 550 °C were repeated twice.^39^ For the multilayer samples, eight cycles of PH_3_ dosing, annealing to 550 °C, and subsequent room temperature deposition of a 1 atom thick Si “locking” layer were performed.^58^ Finally, all dopants were encapsulated by ≈1 nm Si at room temperature and given a short, postdeposition anneal to 350 °C for a few seconds. This triggered a (2 × 1) phase reordering of the Si surface (Figures 3c and d).

### Photoemission Measurements

High-resolution X-ray photoelectron spectroscopy (XPS) measurements of the Si 2p and P 2p core levels were performed throughout the preparation of the single-dosed, double-dosed, and multilayer samples. For each finished structure, the same core levels were subsequently measured using X-ray photoelectron diffraction (XPD). All photoemission measurements were performed at the SuperESCA end station of Elettra Synchrotron in Trieste, Italy. All spectra were collected at room temperature (*T* ≈ 300 K), using a SPECS Phoibos electron energy analyzer equipped with a homemade delay-line detector. The overall energy resolution was Δ*E* < 50 meV for all the measurements. The photoexcitation energies *hν* were calibrated from the kinetic energy difference of Si 2p peaks that were collected using first- and second-order light from the monochromator.

The ultimate concentrations of dopants within the encapsulated δ-layers were estimated by quantitative XPS analysis. Following the P atom incorporation anneal (Figure 3a, I), the surface coverage of P atoms was estimated using a simple two-layer attenuation model.^50^ Next, the intensities of the P 2p subcomponents were tracked during the encapsulation by Si (Figure 3a, II) and the recrystallization of the Si overlayer (Figure 3a, III), fitting each doublet by a pair of symmetric Voigt functions with a spin–orbit energy splitting of 0.84 eV and a 2:1 intensity ratio. Component P1 was shown to originate from the buried δ-layer dopants and thus used to estimate the effective electron carrier density *n* provided (see the Supporting Information for further details^49^). P2 and P3 were interpreted as species near the Si surface.^49,57^ Additionally, a weak component P4 with a disordered spatial structure appeared in the single-dosed case.^49^

XPD patterns from each finished sample were obtained by measuring the Si 2p and P 2p core levels—therein including the P1 subcomponent of the buried δ-layer—over a wide azimuthal sector (φ = 0–130°) and from grazing (θ = 70°) to normal emission (θ = 0°). Each measured spectrum (851 per XPD pattern) was fitted with symmetric Voigt functions to deconvolve the various subcomponents present. Intensity variations between their inelastic backgrounds were also accounted for. Finally, the intensity *I*(θ, φ) of each fitted subcomponent was used to produce polar plots of their modulation functions χ (commonly referred to as “stereographic projections”^36^), defined as
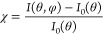
1where *I*_0_(θ) is the average intensity for a given θ across all the azimuthal (φ) scans.^37^

For comparison, X-ray photoelectron diffraction patterns corresponding to different P dopant coordinations were simulated using the Electron Diffraction in Atomic Clusters (EDAC) package.^60^ In the simulations, the atomic origin and angular momentum character of the photoelectron source wave were considered,^59^ the interaction volume around each emitter atom was limited to a radius λ, and the photoemission to a cone with a half-width angle of ≤5° to represent the finite acceptance angle of the photoelectron analyzer.

To determine the atomic coordination of the dopants within the δ-layer structure, the degree of agreement between each measured and simulated diffraction pattern was quantified by a “reliability” factor *R*:
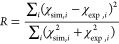
2where χ_exp,*i*_ and χ_sim,*i*_ correspond to the experimental and simulated intensity modulation functions, respectively. The sum index *i* runs over all the available data points at the measured angles. The lower the *R*, the better the agreement between the experiment and the atomic model (*R* = 0 corresponds to a complete agreement; *R* = 1 means no correlation; and *R* = 2 signifies anticorrelation^35^). The best understanding of the atomic arrangement was determined by minimizing *R* upon iterative adjustments of the simulated XPD, with subsequent comparison to the experimental XPD, until an optimum fit between the two was reached. The accuracy of the atomic positions was estimated from Δ*R* ≤ +10% to the minimum *R*-factor.

## Data Availability

The data underpinning the findings presented in this publication can be made available from the corresponding author upon reasonable request.

## Supporting Information Available

Further details of the sample preparation, quantitative and photon energy-dependent XPS analysis, and additional XPD data and modeling of the P dopants, the Si bulk structure, and the Si overlayer (PDF)

## References

[ref1] Zwanenburg F. A.; Dzurak A. S.; Morello A.; Simmons M. Y.; Hollenberg L. C. L.; Klimeck G.; Rogge S.; Coppersmith S. N.; Eriksson M. A. Silicon Quantum Electronics. Rev. Mod. Phys. 2013, 85, 961–1019. 10.1103/RevModPhys.85.961.

[ref2] Veldhorst M.; Eenink H. G. J.; Yang C. H.; Dzurak A. S. Silicon CMOS Architecture for a Spin-Based Quantum Computer. Nat. Commun. 2017, 8, 1766 10.1038/s41467-017-01905-6. 29242497 PMC5730618

[ref3] Gonzalez-Zalba M. F.; de Franceschi S.; Charbon E.; Meunier T.; Vinet M.; Dzurak A. S. Scaling Silicon-Based Quantum Computing Using CMOS Technology. Nat. Electron. 2021, 4, 872–884. 10.1038/s41928-021-00681-y.

[ref4] Sigillito A. J.; Gullans M. J.; Edge L. F.; Borselli M.; Petta J. R. Coherent Transfer of Quantum Information in a Silicon Double Quantum Dot Using Resonant SWAP Gates. npj Quantum Inf. 2019, 5, 110 10.1038/s41534-019-0225-0.

[ref5] Ciriano-Tejel V. N.; Fogarty M. A.; Schaal S.; Hutin L.; Bertrand B.; Ibberson L.; Gonzalez-Zalba M. F.; Li J.; Niquet Y.-M.; Vinet M.; Morton J. J. Spin Readout of a CMOS Quantum Dot by Gate Reflectometry and Spin-Dependent Tunneling. PRX Quantum 2021, 2, 010353 10.1103/PRXQuantum.2.010353.

[ref6] Pauka S. J.; Das K.; Kalra R.; Moini A.; Yang Y.; Trainer M.; Bousquet A.; Cantaloube C.; Dick N.; Gardner G. C.; Manfra M. J.; Reilly D. J. A Cryogenic CMOS Chip for Generating Control Signals for Multiple Qubits. Nat. Electron. 2021, 4, 64–70. 10.1038/s41928-020-00528-y.

[ref7] Fuechsle M.; Miwa J. A.; Mahapatra S.; Ryu H.; Lee S.; Warschkow O.; Hollenberg L. C.; Klimeck G.; Simmons M. Y. A Single-Atom Transistor. Nat. Nanotechnol. 2012, 7, 242–246. 10.1038/nnano.2012.21. 22343383

[ref8] Veldhorst M.; Yang C. H.; Hwang J. C. C.; Huang W.; Dehollain J. P.; Muhonen J. T.; Simmons S.; Laucht A.; Hudson F. E.; Itoh K. M.; Morello A.; Dzurak A. S. A Two-Qubit Logic Gate in Silicon. Nature 2015, 526, 410–414. 10.1038/nature15263. 26436453

[ref9] Schofield S. R.; Curson N. J.; Simmons M. Y.; Rueß F. J.; Hallam T.; Oberbeck L.; Clark R. G. Atomically Precise Placement of Single Dopants in Si. Phys. Rev. Lett. 2003, 91, 136104 10.1103/PhysRevLett.91.136104. 14525322

[ref10] Wyrick J.; Wang X.; Namboodiri P.; Kashid R. V.; Fei F.; Fox J.; Silver R. Enhanced Atomic Precision Fabrication by Adsorption of Phosphine into Engineered Dangling Bonds on H–Si Using STM and DFT. ACS Nano 2022, 16, 19114–19123. 10.1021/acsnano.2c08162. 36317737

[ref11] Fuechsle M.; Mahapatra S.; Zwanenburg F. A.; Friesen M.; Eriksson M. A.; Simmons M. Y. Spectroscopy of Few-Electron Single-Crystal Silicon Quantum Dots. Nat. Nanotechnol. 2010, 5, 502–505. 10.1038/nnano.2010.95. 20495552

[ref12] Weber B.; Mahapatra S.; Ryu H.; Lee S.; Fuhrer A.; Reusch T. C. G.; Thompson D. L.; Lee W. C. T.; Klimeck G.; Hollenberg L. C. L.; Simmons M. Y. Ohm’s Law Survives to the Atomic Scale. Science 2012, 335, 64–67. 10.1126/science.1214319. 22223802

[ref13] Oberbeck L.; Curson N. J.; Hallam T.; Simmons M. Y.; Bilger G.; Clark R. G. Measurement of Phosphorus Segregation in Silicon at the Atomic Scale Using Scanning Tunneling Microscopy. Appl. Phys. Lett. 2004, 85, 1359–1361. 10.1063/1.1784881.

[ref14] Goh K. E. J.; Oberbeck L.; Simmons M. Y.; Hamilton A. R.; Clark R. G. Effect of Encapsulation Temperature on Si:P Delta-Doped Layers. Appl. Phys. Lett. 2004, 85, 4953–4955. 10.1063/1.1827940.

[ref15] Goh K. E. J.; Simmons M. Y. Impact of Si Growth Rate on Coherent Electron Transport in Si:P Delta-Doped Devices. Appl. Phys. Lett. 2009, 95, 142104 10.1063/1.3245313.

[ref16] Drumm D. W.; Hollenberg L. C. L.; Simmons M. Y.; Friesen M. Effective Mass Theory of Monolayer δ Doping in the High-Density Limit. Phys. Rev. B 2012, 85, 155419 10.1103/PhysRevB.85.155419.

[ref17] McKibbin S. R.; Clarke W. R.; Simmons M. Y. Investigating the Surface Quality and Confinement of Si:P δ-Layers at Different Growth Temperatures. Physica E Low Dimens. Syst. Nanostruct. 2010, 42, 1180–1183. 10.1016/j.physe.2009.11.111.

[ref18] Polley C. M.; Clarke W. R.; Miwa J. A.; Simmons M. Y.; Wells J. W. Microscopic Four-Point-Probe Resistivity Measurements of Shallow, High Density Doping Layers in Silicon. Appl. Phys. Lett. 2012, 101, 262105 10.1063/1.4773485.

[ref19] Miwa J. A.; Hofmann Ph.; Simmons M. Y.; Wells J. W. Direct Measurement of the Band Structure of a Buried Two-Dimensional Electron Gas. Phys. Rev. Lett. 2013, 110, 136801 10.1103/PhysRevLett.110.136801. 23581353

[ref20] Miwa J. A.; Warschkow O.; Carter D. J.; Marks N. A.; Mazzola F.; Simmons M. Y.; Wells J. W. Valley Splitting in a Silicon Quantum Device Platform. Nano Lett. 2014, 14, 1515–1519. 10.1021/nl404738j. 24571617

[ref21] Mazzola F.; Polley C. M.; Miwa J. A.; Simmons M. Y.; Wells J. W. Disentangling Phonon and Impurity Interactions in δ-Doped Si(001). Appl. Phys. Lett. 2014, 104, 173108 10.1063/1.4874651.

[ref22] Hagmann J. A.; Wang X.; Namboodiri P.; Wyrick J.; Murray R.; Stewart M. D. Jr.; Silver R. M.; Richter C. A. High Resolution Thickness Measurements of Ultrathin Si:P Monolayers Using Weak Localization. Appl. Phys. Lett. 2018, 112, 043102 10.1063/1.4998712.

[ref23] Wang X.; Hagmann J. A.; Namboodiri P.; Wyrick J.; Li K.; Murray R. E.; Myers A.; Misenkosen F.; Stewart M. D.; Richter C. A.; Silver R. M. Quantifying Atom-Scale Dopant Movement and Electrical Activation in Si:P Monolayers. Nanoscale 2018, 10, 4488–4499. 10.1039/C7NR07777G. 29459919 PMC11305481

[ref24] Holt A. J.; Mahatha S. K.; Stan R.-M.; Strand F. S.; Nyborg T.; Curcio D.; Schenk A. K.; Cooil S. P.; Bianchi M.; Wells J. W.; Hofmann Ph.; Miwa J. A. Observation and Origin of the Δ Manifold in Si:P δ Layers. Phys. Rev. B 2020, 101, 121402 10.1103/PhysRevB.101.121402.

[ref25] Mazzola F.; Chen C.-Y.; Rahman R.; Zhu X.-G.; Polley C. M.; Balasubramanian T.; King P. D.; Hofmann Ph.; Miwa J. A.; Wells J. W. The Sub-Band Structure of Atomically Sharp Dopant Profiles in Silicon. npj Quantum Mater. 2020, 5, 1–5. 10.1038/s41535-020-0237-1.

[ref26] Ivie J. A.; Campbell Q.; Koepke J. C.; Brickson M. I.; Schultz P. A.; Muller R. P.; Mounce A. M.; Ward D. R.; Carroll M. S.; Bussmann E.; Baczewski A. D.; Misra S. Impact of Incorporation Kinetics on Device Fabrication with Atomic Precision. Phys. Rev. Appl. 2021, 16, 054037 10.1103/PhysRevApplied.16.054037.

[ref27] Carter D. J.; Warschkow O.; Marks N. A.; McKenzie D. R. Electronic Structure Models of Phosphorus δ-Doped Silicon. Phys. Rev. B 2009, 79, 033204 10.1103/PhysRevB.79.033204.

[ref28] Lee S.; Ryu H.; Campbell H.; Hollenberg L. C. L.; Simmons M. Y.; Klimeck G. Electronic Structure of Realistically Extended Atomistically Resolved Disordered Si:P δ-Doped Layers. Phys. Rev. B 2011, 84, 205309 10.1103/PhysRevB.84.205309.

[ref29] Carter D. J.; Marks N. A.; Warschkow O.; McKenzie D. R. Phosphorus δ-Doped Silicon: Mixed-Atom Pseudopotentials and Dopant Disorder Effects. Nanotechnology 2011, 22, 065701 10.1088/0957-4484/22/6/065701. 21212477

[ref30] Chubarov M.; Choudhury T. H.; Zhang X.; Redwing J. M. In-Plane X-Ray Diffraction for Characterization of Monolayer and Few-Layer Transition Metal Dichalcogenide Films. Nanotechnology 2018, 29, 055706 10.1088/1361-6528/aaa1bd. 29239306

[ref31] Yamashita S.; Kikkawa J.; Yanagisawa K.; Nagai T.; Ishizuka K.; Kimoto K. Atomic Number Dependence of Z Contrast in Scanning Transmission Electron Microscopy. Sci. Rep. 2018, 8, 1–7. 10.1038/s41598-018-30941-5. 30120323 PMC6098135

[ref32] Young S. M.; Katzenmeyer A. M.; Anderson E. M.; Luk T. S.; Ivie J. A.; Schmucker S. W.; Gao X.; Misra S. Suppression of Mid-Infrared Plasma Resonance due to Quantum Confinement in Delta-Doped Silicon. 2022; https://arxiv.org/abs/2210.10711.

[ref33] Hüfner S. Photoelectron Spectroscopy: Principles and Applications; Springer Science & Business Media: Berlin, Germany, 2013; Chapter 11.

[ref34] Bengió S.; Wells J. W.; Kim T. K.; Zampieri G.; Petaccia L.; Lizzit S.; Hofmann Ph. The Structure of Sb(111) Determined by Photoelectron Diffraction. Surf. Sci. 2007, 601, 2908–2911. 10.1016/j.susc.2007.04.251.

[ref35] Woodruff D. Adsorbate Structure Determination Using Photoelectron Diffraction: Methods and Applications. Surf. Sci. Rep. 2007, 62, 1–38. 10.1016/j.surfrep.2006.10.001.

[ref36] Bignardi L.; Lizzit D.; Bana H.; Travaglia E.; Lacovig P.; Sanders C. E.; Dendzik M.; Michiardi M.; Bianchi M.; Ewert M.; Buß L.; Falta J.; Flege J. I.; Baraldi A.; Larciprete R.; Hofmann Ph.; Lizzit S. Growth and Structure of Singly Oriented Single-Layer Tungsten Disulfide on Au(111). Phys. Rev. Mater. 2019, 3, 014003 10.1103/PhysRevMaterials.3.014003.

[ref37] Holt A. J. U.; Pakdel S.; Rodríguez-Fernández J.; Zhang Y.; Curcio D.; Sun Z.; Lacovig P.; Yao Y.-X.; Lauritsen J. V.; Lizzit S.; Lanatà N.; Hofmann Ph.; Bianchi M.; Sanders C. E. Electronic Properties of Single-Layer CoO_2_/Au(111). 2D Mater. 2021, 8, 035050 10.1088/2053-1583/ac040f.

[ref38] McKibbin S. R.; Clarke W. R.; Fuhrer A.; Reusch T. C. G.; Simmons M. Y. Investigating the Regrowth Surface of Si:P δ-Layers Toward Vertically Stacked Three Dimensional Devices. Appl. Phys. Lett. 2009, 95, 233111 10.1063/1.3269924.

[ref39] McKibbin S.; Polley C.; Scappucci G.; Keizer J.; Simmons M. Low Resistivity, Super-Saturation Phosphorus-in-Silicon Monolayer Doping. Appl. Phys. Lett. 2014, 104, 123502 10.1063/1.4869111.

[ref40] Wang Y.; Bronikowski M. J.; Hamers R. J. An Atomically Resolved STM Study of the Interaction of Phosphine With the Silicon (001) Surface. J. Phys. Chem. 1994, 98, 5966–5973. 10.1021/j100074a025.

[ref41] Wilson H. F.; Warschkow O.; Marks N. A.; Schofield S. R.; Curson N. J.; Smith P. V.; Radny M. W.; McKenzie D. R.; Simmons M. Y. Phosphine Dissociation on the Si(001) Surface. Phys. Rev. Lett. 2004, 93, 226102 10.1103/PhysRevLett.93.226102. 15601102

[ref42] Curson N. J.; Schofield S. R.; Simmons M. Y.; Oberbeck L.; O’Brien J. L.; Clark R. G. STM Characterization of the Si-P Heterodimer. Phys. Rev. B 2004, 69, 195303 10.1103/PhysRevB.69.195303.

[ref43] Warschkow O.; Wilson H. F.; Marks N. A.; Schofield S. R.; Curson N. J.; Smith P. V.; Radny M. W.; McKenzie D. R.; Simmons M. Y. Phosphine Adsorption and Dissociation on the Si(001) Surface: An Ab Initio Survey of Structures. Phys. Rev. B 2005, 72, 125328 10.1103/PhysRevB.72.125328. 15601102

[ref44] Song F.; Monsen Å.; Li Z. S.; Choi E.-M.; MacManus-Driscoll J. L.; Xiong J.; Jia Q. X.; Wahlström E.; Wells J. W. Extracting the Near Surface Stoichiometry of BiFe_0.5_Mn_0.5_O_3_ Thin Films; a Finite Element Maximum Entropy Approach. Surf. Sci. 2012, 606, 1771–1776. 10.1016/j.susc.2012.06.016.

[ref45] Mazzola F.; Edmonds M. T.; Høydalsvik K.; Carter D. J.; Marks N. A.; Cowie B. C.; Thomsen L.; Miwa J.; Simmons M. Y.; Wells J. W. Determining the Electronic Confinement of a Subsurface Metallic State. ACS Nano 2014, 8, 10223–10228. 10.1021/nn5045239. 25243326

[ref46] Cooil S. P.; Mazzola F.; Klemm H. W.; Peschel G.; Niu Y. R.; Zakharov A. A.; Simmons M. Y.; Schmidt T.; Evans D. A.; Miwa J. A.; Wells J. W. In Situ Patterning of Ultrasharp Dopant Profiles in Silicon. ACS Nano 2017, 11, 1683–1688. 10.1021/acsnano.6b07359. 28182399

[ref47] Wider J.; Greber T.; Wetli E.; Kreutz T. J.; Schwaller P.; Osterwalder J. Direct Observation of Subsurface Oxygen on Rh(111). Surf. Sci. 1998, 417, 301–310. 10.1016/S0039-6028(98)00674-8.

[ref48] Pillo T.; Hayoz J.; Schwaller P.; Berger H.; Aebi P.; Schlapbach L. Substitution Sites of Pb and Y in Bi_2_Sr_2_Ca_1_Cu_2_O_8+δ_: X-Ray Photoelectron Diffraction as Fingerprinting Tool. Appl. Phys. Lett. 1999, 75, 1550–1552. 10.1063/1.124751.

[ref49] See the Supporting Information for further details of the sample preparation, quantitative and photon energy-dependent XPS analysis, and additional XPD data and modeling of the P dopants, Si bulk, and Si overlayer.

[ref50] Røst H. I.; Chellappan R. K.; Strand F. S.; Grubišić-Čabo A.; Reed B. P.; Prieto M. J.; Tǎnase L. C.; de Souza Caldas L.; Wongpinij T.; Euaruksakul C.; Schmidt T.; Tadich A.; Cowie B. C. C.; Li Z.; Cooil S. P.; Wells J. W. Low-Temperature Growth of Graphene on a Semiconductor. J. Phys. Chem. C 2021, 125, 4243–4252. 10.1021/acs.jpcc.0c10870.

[ref51] Kittel C.; McEuen P. Introduction to Solid State Physics, 8th ed.; John Wiley & Sons: New York, 2018; Chapter 1.

[ref52] Seah M. P.; Dench W. A. Quantitative Electron Spectroscopy of Surfaces: A Standard Data Base for Electron Inelastic Mean Free Paths in Solids. Surf. Interface Anal. 1979, 1, 2–11. 10.1002/sia.740010103.

[ref53] Bana H.; Travaglia E.; Bignardi L.; Lacovig P.; Sanders C. E.; Dendzik M.; Michiardi M.; Bianchi M.; Lizzit D.; Presel F.; De Angelis D.; Apostol N.; Das P. K.; Fujii J.; Vobornik I.; Larciprete R.; Baraldi A.; Hofmann Ph.; Lizzit S. Epitaxial Growth of Single-Orientation High-Quality MoS_2_ Monolayers. 2D Mater. 2018, 5, 035012 10.1088/2053-1583/aabb74.

[ref54] Ramstad A.; Brocks G.; Kelly P. J. Theoretical Study of the Si(100) Surface Reconstruction. Phys. Rev. B 1995, 51, 14504–14523. 10.1103/PhysRevB.51.14504. 9978383

[ref55] Tang S.; Freeman A. J.; Delley B. Structure of the Si(100) 2 × 1 Surface: Total-Energy and Force Analysis of the Dimer Models. Phys. Rev. B 1992, 45, 1776–1783. 10.1103/PhysRevB.45.1776. 10001679

[ref56] Tsukidate Y.; Suemitsu M. Saturated Adsorption of PH_3_ on Si(100):P and its Application to Digital Control of Phosphorus Coverage on Si(100) Surface. Appl. Surf. Sci. 1999, 151, 148–152. 10.1016/S0169-4332(99)00272-X.

[ref57] Wilson H. F.; Warschkow O.; Marks N. A.; Curson N. J.; Schofield S. R.; Reusch T. C. G.; Radny M. W.; Smith P. V.; McKenzie D. R.; Simmons M. Y. Thermal Dissociation and Desorption of PH_3_ on Si(001): A Reinterpretation of Spectroscopic Data. Phys. Rev. B 2006, 74, 195310 10.1103/PhysRevB.74.195310.

[ref58] Keizer J. G.; McKibbin S. R.; Simmons M. Y. The Impact of Dopant Segregation on the Maximum Carrier Density in Si:P Multilayers. ACS Nano 2015, 9, 7080–7084. 10.1021/acsnano.5b01638. 26083628

[ref59] Greber T.; Osterwalder J.; Naumović D.; Stuck A.; Hüfner S.; Schlapbach L. Auger Electron and Photoelectron Angular Distributions from Surfaces: Importance of the Electron Source Wave. Phys. Rev. Lett. 1992, 69, 1947–1950. 10.1103/PhysRevLett.69.1947. 10046357

[ref60] García de Abajo F. J.; Van Hove M. A.; Fadley C. S. Multiple Scattering of Electrons in Solids and Molecules: A Cluster-Model Approach. Phys. Rev. B 2001, 63, 075404 10.1103/PhysRevB.63.075404.

[ref61] Ramanayaka A. N.; Kim H.-S.; Hagmann J. A.; Murray R. E.; Tang K.; Meisenkothen F.; Zhang H. R.; Bendersky L. A.; Davydov A. V.; Zimmerman N. M.; Richter C. A.; Pomeroy J. M. Towards Superconductivity in p-Type Delta-Doped Si/Al/Si Heterostructures. AIP Adv. 2018, 8, 075329 10.1063/1.5045338.

[ref62] Stock T. J. Z.; Warschkow O.; Constantinou P. C.; Li J.; Fearn S.; Crane E.; Hofmann E. V. S.; Kölker A.; McKenzie D. R.; Schofield S. R.; Curson N. J. Atomic-Scale Patterning of Arsenic in Silicon by Scanning Tunneling Microscopy. ACS Nano 2020, 14, 3316–3327. 10.1021/acsnano.9b08943. 32142256 PMC7146850

[ref63] Dwyer K. J.; Baek S.; Farzaneh A.; Dreyer M.; Williams J. R.; Butera R. E. B-Doped δ-Layers and Nanowires from Area-Selective Deposition of BCl_3_ on Si(100). ACS Appl. Mater. Interfaces 2021, 13, 41275–41286. 10.1021/acsami.1c10616. 34405671

