# Supplementary material for: Probing the Atomic Arrangement of Subsurface Dopants in a Silicon Quantum Device Platform
Source: ACS Appl Mater Interfaces. 2023 Apr 28;15(18):22637–43. doi: 10.1021/acsami.2c23011 (PMC10176322; doi:10.1021/acsami.2c23011)
Supplement: Supplementary file 1 — am2c23011_si_001.pdf [file am2c23011_si_001.pdf]

Supporting Information: Probing the Atomic Arrangement of Subsurface Dopants in a Silicon Quantum Device Platform

Håkon I. Røst,[1,2] Ezequiel Tosi,[3,4] Frode S. Strand,[1] Anna Cecilie Åsland,[1] Paolo Lacovig,[3] Silvano Lizzit,[3] and Justin W. Wells[1,5,*]

[1] *Center for Quantum Spintronics, Department of Physics, Norwegian University of Science and Technology (NTNU), NO-7491 Trondheim, Norway.*

[2] *Department of Physics and Technology, University of Bergen (UiB), Allégaten 55, 5007 Bergen, Norway.*

[3] *Elettra - Sincrotrone Trieste, s.s. 14 - km.163, 5 in Area Science Park, Basovizza, Trieste, 34149, Italy.*

[4] *Instituto de Ciencia de Materiales de Madrid (ICMM-CSIC) C/ Sor Juana Inés de la Cruz 3, 28049, Madrid, Spain.*

[5] *Department of Physics and Centre for Materials Science and Nanotechnology, University of Oslo (UiO), Oslo 0318, Norway.*

S1. SURFACE PH_3 ADSORPTION AND P DOPANT INCORPORATION

At room temperature ($T \approx 300\,\mathrm{K}$), gaseous phosphine is known to dissociate and bond with the Si−Si dimers on Si(001), following a pathway of three successive H deficient products with different reaction rates [1]. In a manner of minutes, fractions of the adsorbed gas rapidly dissociate from $PH_3 \rightarrow PH_2 + H$, then $PH_2 \rightarrow PH + H$, and finally $PH \rightarrow P + H$ [2, 3]. The result is a mixture of PH_2, PH, and P on the surface, the former two products having non-negligible energy barriers to further decompose. If annealed above 650 K, H_2 gas is released and the P atoms incorporated into the surface by substituting for Si [4–6].

An X-ray photoelectron spectroscopy (XPS) study of room temperature adsorption and dissociation of phosphine gas onto Si(001) and subsequent, thermally promoted surface incorporation of P atoms is shown in FIG. S1. Upon adsorption, two leading doublets can be distinguished from the P 2p core level in the range 133-135 eV, along with a faint broad feature at lower binding energies. Based on the previous XPS studies by Lin *et al.* and their re-interpretation by Wilson *et al.*, the two main doublets can be assigned to surface-bound PH (133.57 eV) and PH_2 (134.15 eV) [1, 7]. The origin of the final component P3 is unclear, but it is assumed to be PH_x at a different, less energetically favorable binding site (see e.g. FIG. 2 of Ref. 3). The Si 2p core level has four doublets: three are known bulk- (B, 103.58 eV) and surface-related (S', 103.82 eV; S, 103.40 eV) components [8], and the final one matches with Si^+ from a minuscule amount of intermediary Si_2O oxide (104.29 eV) [9].

Upon thermal activation at 820 K the PH and PH_2 signals depleted, and a new leading feature P2 appeared at 133.20 eV. A similar change was observed by Lin *et al.* (FIG. 3b, Ref. 7) and can be attributed to the incorporation of P atoms into the Si surface [1]. P3 was still present but in higher quantity, and a new feature P1 appeared at 134.80 eV, i.e., far away from any of the pre-existing dissociation components. This latter feature can be assigned to P dopants beneath the Si surface, as made evident from the discussions in the main paper and the energy-dependent XPS in Section S3.

* Corresponding author: j.w.wells@fys.uio.no

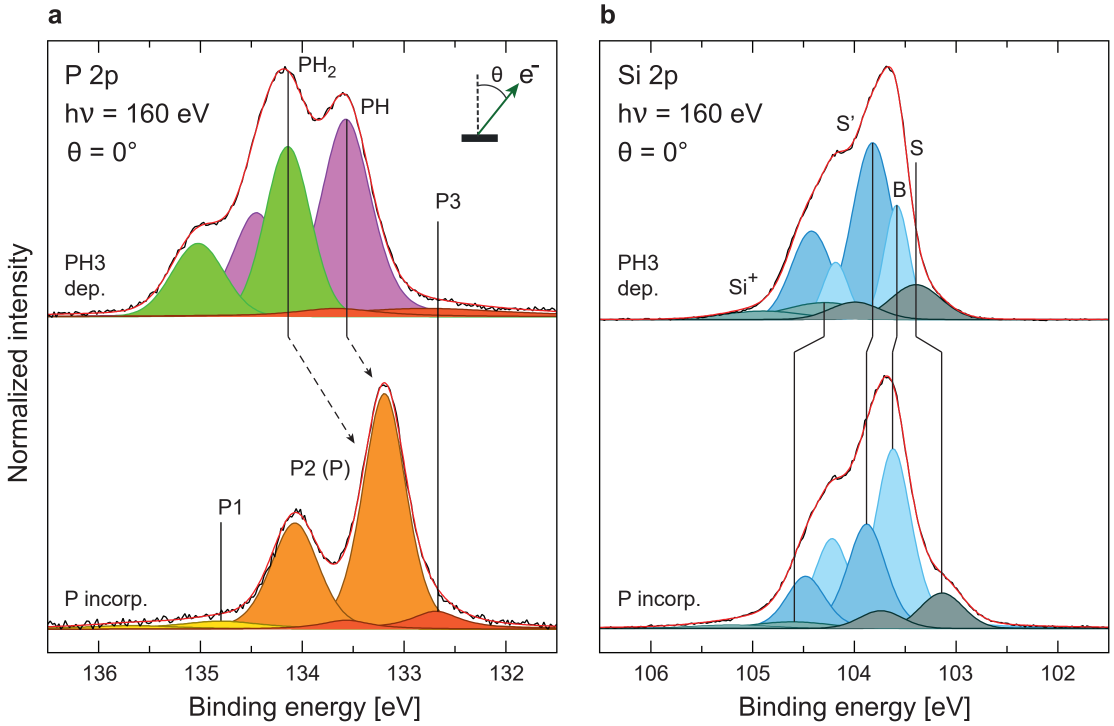

FIG. S1. Normal emission ($\theta = 0°$) XPS of P 2p and Si 2p after PH_3 adsorption and subsequent P dopant incorporation into Si(001). **a**: Components PH_2, PH and P3 are dissociation products of adsorbed PH_3, while P2 and P1 are P dopants in and beneath the Si(100) surface, respectively [2, 7]. **b**: Component B stems from bulk Si while S' and S are from surface Si dimers and the second layer atoms, respectively [8]. The tiny Si^+ is from remnants of intermediary oxide at the surface [9]. All spectra have been normalized to the maximum peak intensity of each core level.

S2. QUANTIFYING THE P DOPANT DENSITY WITHIN THE δ-LAYER

The coverage of P atoms on a Si(001) surface after PH_3 adsorption, dissociation, and incorporation can be estimated from the relative intensities of the Si 2p and P 2p core levels using a simple two-layer attenuation model [10]. In our experiments, a single exposure of 1.125 Langmuirs (L) PH_3 at 300 K and a subsequent, rapid annealing to 820 K resulted in a 0.17 monolayer (ML) surface coverage (FIG. S1). Repeating the dosing and rapid annealing a second time (i.e., 'double-dosing') yielded a total surface coverage of 0.39 ML.

Following the incorporation of P atoms, each system was studied in real-time by fast-XPS as their doped surfaces were encapsulated by depositing a $\approx$ 1 nm Si overlayer [11]. From each measured P 2p spectrum, the individual contributions from components P1, P2,

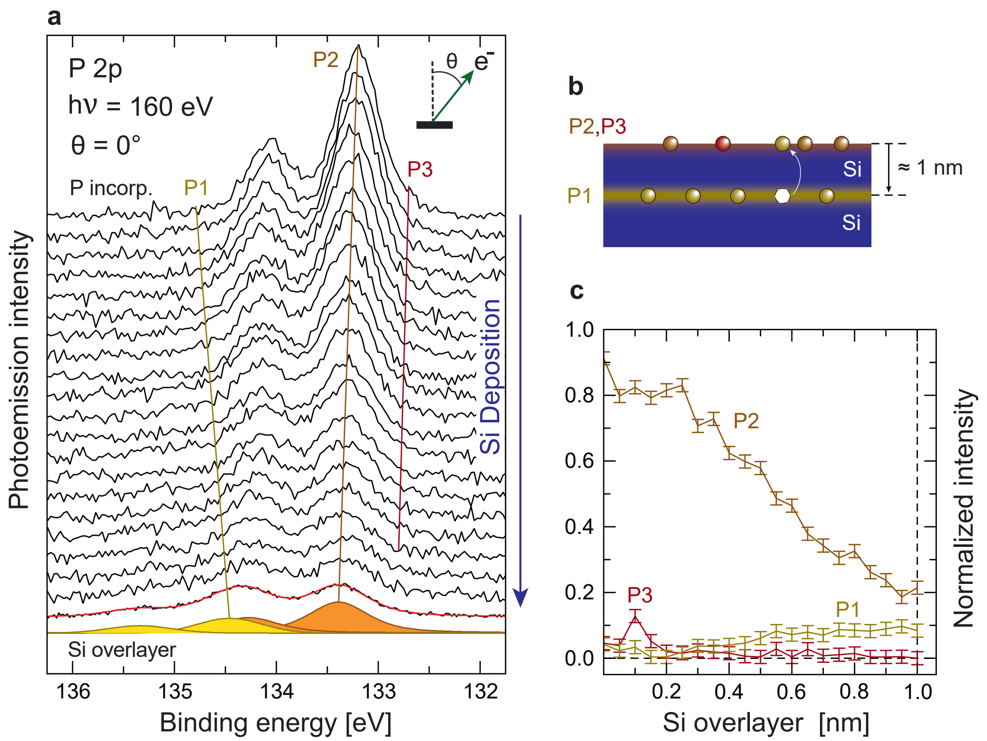

FIG. S2. The evolution of the P 2p core level measured from a 'single-dosed' δ-layer sample during Si overlayer growth **a**: Fast-XPS spectra of P 2p, measured at normal emission ($\theta = 0°$) during the deposition of a 1 nm thick Si overlayer at room temperature. The deconvolved sub-components of the final spectrum have also been shown. **b**: A sketch showing the measured layer structure of a single-dosed Si:P δ-layer. **c**: P 2p sub-component intensities as a function of Si overlayer thickness, normalized to the initial intensity measured at 0 nm of deposited Si.

and P3 to were quantified. An example of such analysis from our 'single-dosed' δ-layer sample is shown in FIG. S2. The surface P2 and the total P 2p intensities were observed to reduce linearly vs. time (FIG. S2**c**), i.e., contrary to the negative exponential intensity decay expected for encapsulated layers [12]. Also, the P1 intensity grew in absolute terms and the P3 intensity was completely diminished.

Additional, angle- and energy-dependent XPS measurements of the grown single-dosed, double-dosed, and multilayer Si:P δ-layer structures revealed that in each case, (i) the components P2 and P3 were still situated at the sample surface after the Si encapsulation, and (ii) that P1 belonged to the buried δ-layer (FIG. S2**b**, see Section S3 for details). Hence by assuming a constant, total amount of P atoms present in the system, and furthermore that P2 and P3 had been located at the surface at all times during the Si deposition,

the additional P1 formed could be estimated from the loss of surface P 2p intensity as $-\Delta\text{P2} - \Delta\text{P3} = +\Delta\text{P1}$. A final re-crystallization anneal ($T \approx 620$ K) of each finished structure promoted some additional segregation of P to the surface, thus increasing the intensities of P2 and P3 in absolute terms [13–15]. The P1 was shown to remain in the δ-layer. Also, an additional component P4 appeared for the single-dosed samples only, exhibiting a disordered spatial structure (as determined by XPD measurements, see Section S6).

By tracing the development of the P1 intensities throughout the growth steps and comparing the resultant ML coverages of their corresponding δ-layers to the atomic sheet density of the Si(001) plane the added, effective electron carrier density n to each system could be estimated. For single- and double-dosing, the final δ-layer sheet densities achieved were approximately 0.08 ML and 0.34 ML, respectively. Their resulting free carrier densities were thus $n_{\text{single}} = 5.1 \times 10^{13}$ cm^{-2} and $n_{\text{double}} = 2.3 \times 10^{14}$ cm^{-2}. Both values are well above the metal-to-insulator transition [16], and roughly consistent with previous reports [6, 7, 17, 18].

S3. PROBING THE SI-ENCAPSULATED P DOPANTS WITH INCREASING λ

In photoemission experiments, the kinetic energy ε dependence of the inelastic mean-free path $\lambda(\varepsilon)$ for outgoing photoelectrons can be used to determine the layer structure of a system. On average, $\lambda(\varepsilon) \propto \varepsilon^{-2}$ for low kinetic energies ($\varepsilon \leq 30\,\text{eV}$), while for higher kinetic energies ($\varepsilon > 75\,\text{eV}$) then $\lambda(\varepsilon) \propto \varepsilon^{1/2}$ [19]. Changing ε will thus change the average effective escape depth of photoelectrons from beneath the surface of a material.

A simple, yet effective attenuation model for photoelectron intensity can be constructed from the Beer-Lambert law as

$$I(d, \theta) \propto I_0 \exp\{-d/[\lambda(\varepsilon)\cos\theta]\}, \tag{S1}$$

where d is the probing depth in the direction orthogonal to the surface, θ the emission angle relative to the surface normal, and I_0 the signal intensity from the surface ($d = 0$) [12]. According to the model, signals from features at larger d should attenuate exponentially and also roll off more rapidly with θ than signals originating from the surface layers. Furthermore, the attenuation can be controlled by changing the ratio $d/\lambda(\varepsilon)$. For instance, the detectable signal from features that are spatially localized along d, e.g. an atomically thin, encapsulated δ-layer, can be either enhanced or diminished when $\lambda(\varepsilon)$ is varied.

The measured P 2p signal from a double-dosed δ-layer is shown in FIG. S3**a** as a func-

tion of increasing $\lambda(\varepsilon)$. Notably, at larger $\lambda(\varepsilon)$ the intensities of P1 and P3 increased and decreased relative to P2, respectively. As discussed in Section S1, P2 can be assigned to the incorporated P dopants near the surface. The relative increase in P1, therefore, suggests that at larger $\lambda(\varepsilon)$, more P1 signal is added into the increasingly thicker slab of excitation volume beneath the surface. This matches with the P1 signal originating from the dopants in the δ-layer encapsulated in Si. Additionally, the relatively decreasing P3 intensity suggests that this specie was local to the Si surface, perhaps even more so than P2.

S4. ATOMIC MODELS FOR P DOPANT PLACEMENT IN SI

Different XPD patterns expected from different P dopant configurations were simulated for comparison with the measured X-ray photoelectron diffraction (XPD) from the P 2p core level (FIGs. 1-2, main text), using the Electron Diffraction in Atomic Clusters (EDAC) package [21]. In FIGs. S3**b-g**, sketches of the atomic models used for the dopant placement, and their simulated XPD patterns compared to the measured P1 XPD pattern, are shown.

To model substitutional doping, Si atoms in the fcc 'diamond'-like unit cell were replaced by P atoms one by one, i.e. so that each possible P dopant position would neighbor with Si atoms. The resulting XPD pattern for each inequivalent electron emitter (P atom) position was then simulated. Finally, all contributions (9 in total) were averaged to form the resultant, total XPD pattern. In the case of P−P dimerization, adjacent Si atoms in the Si(001) plane got replaced by P atoms and subsequently relaxed towards each other to form P−P bonds along the [110] direction. An optimum bond length of $d = 2.42\,\text{Å}$ was found, i.e., slightly longer than the bulk Si−Si bond length (2.35 Å) [22], and an intermediate value between other reported P−P bond lengths [23]. For the cluster model, all 9 inequivalent Si atoms in the unit cell were simultaneously replaced by P atoms, retaining the same nearest-neighbor bond length as that of bulk Si.

At $h\nu = 250\,\text{eV}$ ($\varepsilon \approx 115\,\text{eV}$) the measured XPD signal from the P dopants (P1) achieved a moderate agreement with the substitutional and cluster-like doping models ($R = 0.67$ and $R = 0.63$, respectively). The P−P dimer model ($R = 1.21$) showed no correlation with the measurements and was therefore ruled out completely. Having already established the total doping concentration and also the confined location of the P dopants beneath the Si surface (FIG. S3**a**, and FIG. 1**a** in the main text), the high-concentration cluster-like doping could also be ruled out. Hence substitutional doping was the only likely scenario.

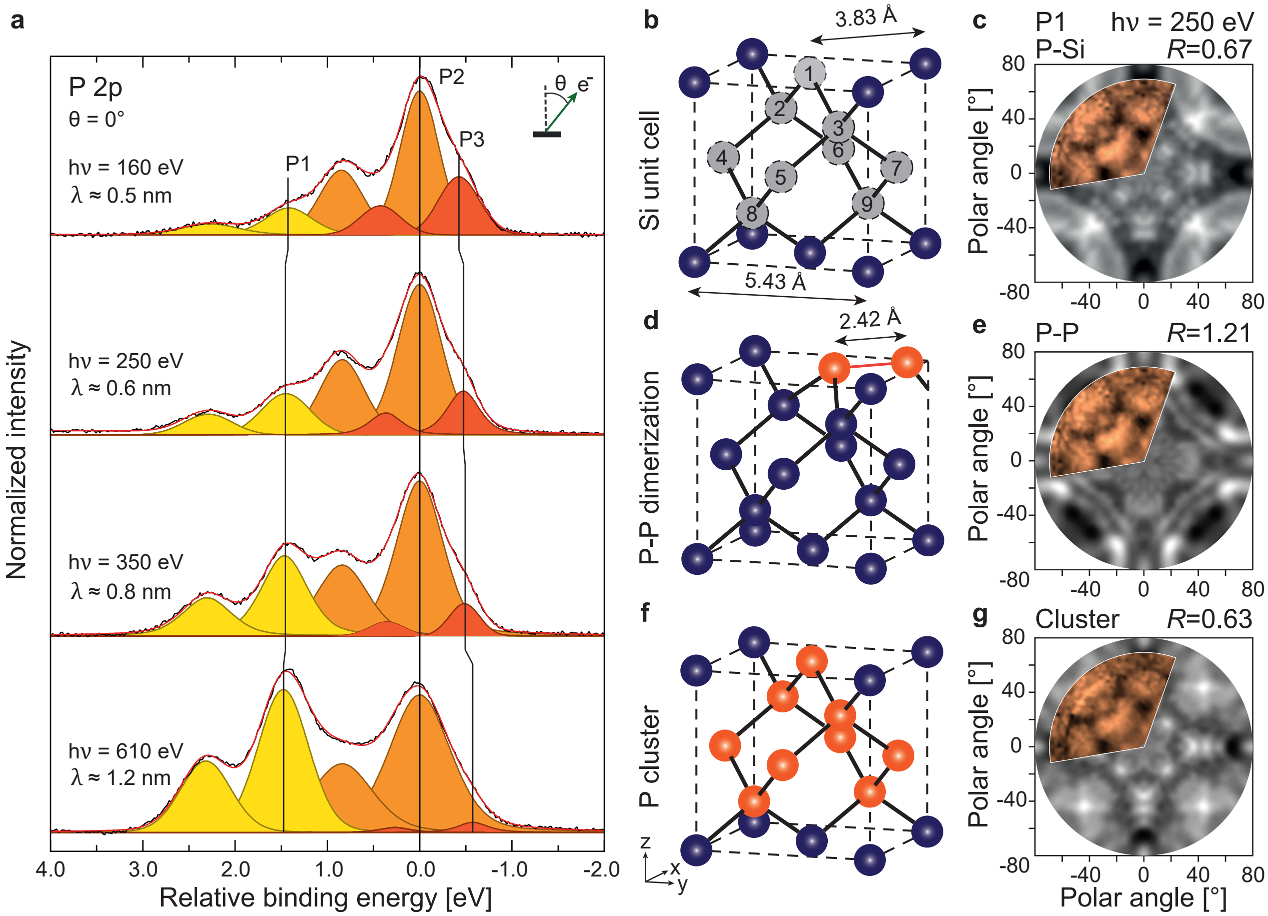

FIG. S3. Energy-dependent P 2p core level analysis and modeling of P dopant atomic placement. **a**: The measured P 2p signal at normal emission from a double-dosed δ-layer system as a function of increasing photoelectron kinetic energy ε and mean-free path $\lambda(\varepsilon)$. The core levels have been plotted on a binding energy scale relative to the P2 feature ($E_{\mathrm{B}} = 133.20\,\mathrm{eV}$). All spectra have been normalized to the measured peak maximum intensity. **b**–**g**: Lattice models for P atom placements in δ-doped Si and their corresponding, simulated XPD patterns. In the Si fcc unit cell [20], there are 9 inequivalent atomic positions. For substitutional doping (**b**, **c**), either site can be occupied by a P atom and will have Si atoms as its neighbors. For in-plane P−P dimerization (**d**, **e**), neighboring Si atoms in the (001) plane were replaced and the newfound P atoms displaced in-plane to form bonds. In the cluster model (**f**, **g**), all 9 inequivalent Si atoms got replaced simultaneously.

S5. SIMULATING XPD WITH THE CORRECT SI SURFACE STRUCTURE

For comparison with the more surface-sensitive XPD measurements ($h\nu = 220 - 250\,\mathrm{eV}$), the Si lattice model was adjusted to account for the observed (2×1) reconstruction at the surface of the encapsulation layer (see FIG. 3**d** in the main text). Using a crude model that ignored any relaxation between the topmost atomic layers, the Si atoms in the surface layer were perturbed towards each other by a distance Δa [Å] (FIG. S4**a**), yielding an optimal reliability factor $R = 0.23$ at $\Delta a = 0.3\,\text{Å}$ (FIGs. S4**c**, S4**d**). Note that this displacement is too small for a proper Si−Si dimerization to occur [20, 22]. Therefore, it merely served as a first step toward a more accurate surface model. In contrast, a negligible Δa was preferred to minimize R when comparing with measurements of photoelectrons with higher kinetic energies ε ($h\nu = 320 - 350\,\mathrm{eV}$, FIGs. S4**b**, S4**c**). This can be explained by the expected reduction in electron scattering along other directions than the surface normal at larger ε, and also the increased bulk sensitivity achieved with an increased photoelectron mean-free path $\lambda(\varepsilon)$ [19, 24].

S6. XPD OF THE P4 COMPONENT FROM THE SINGLE-DOSED SYSTEM

In FIG. S5, XPD patterns of the P4 sub-component of the P 2p signal as measured from a single-dosed δ-layer sample are shown. With the relatively surface-sensitive photoexcitation energy used ($h\nu = 250\,\mathrm{eV}$), the P4 photoelectrons were ejected with a $\varepsilon \approx 115\,\mathrm{eV}$. As shown in FIG. S5**a**, only a faint ordering is visible that is vaguely reminiscent of the one observed from P1 and bulk Si. When compared with the corresponding, optimized XPD simulation for the bulk Si structure at a similar ε (see the previous Section), a reliability factor of $R = 0.88$ was achieved. In other words, there was little agreement between the measured and the calculated XPD patterns. Interestingly, the R-factor is seen to improve at larger ε away from the peak, i.e., when approaching the kinetic energy of the P1 signal originating from the buried δ-layer (FIGs. S5**b**- S5**c**). The reason for this is unclear, but may perhaps suggest that the species P1 and P4 have some similarities in their atomic arrangements. Ultimately, no strong evidence for any crystalline order for the P4 species was observed.

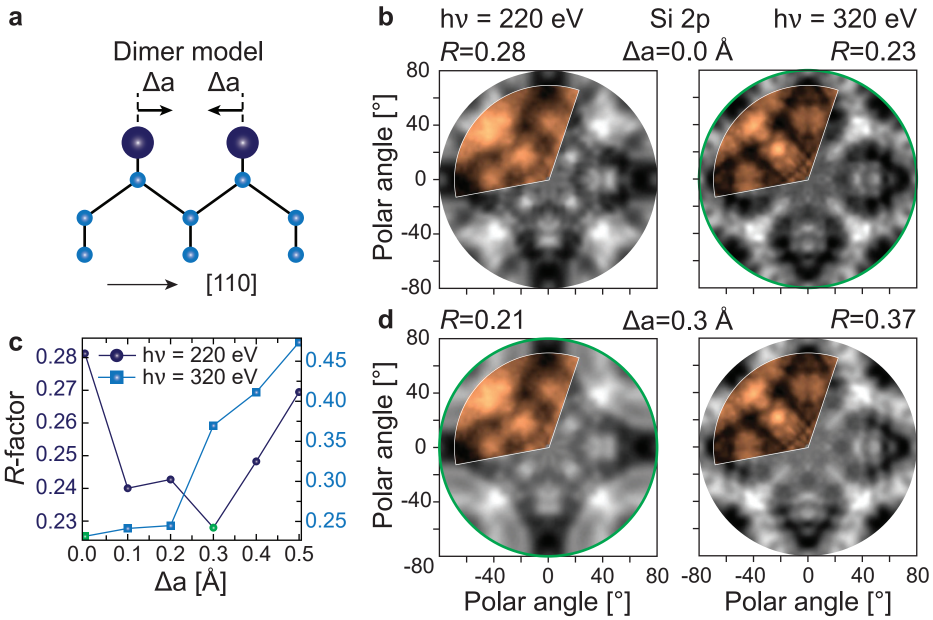

FIG. S4. Si surface dimerization and kinetic energy-dependent XPD measurements of Si:P δ-layers. **a**: A side view, ball-and-stick model of a dimerizing Si(001) surface along the [110] direction. **b**–**d**: Reliability (R) factor optimization for Si 2p ('B') photoelectrons excited with different photon energies $h\nu$, as a function of Si surface atom displacement Δa towards dimerization (**c**). Comparisons of the measured (orange) and simulated (grey) XPD patterns, shown for two different displacements Δa with the best fit at each $h\nu$ circled in green (**b**, **d**). The measurements at $h\nu = 220\,\text{eV}$ and $h\nu = 320\,\text{eV}$ were from a single-dosed and double-dosed system, respectively.

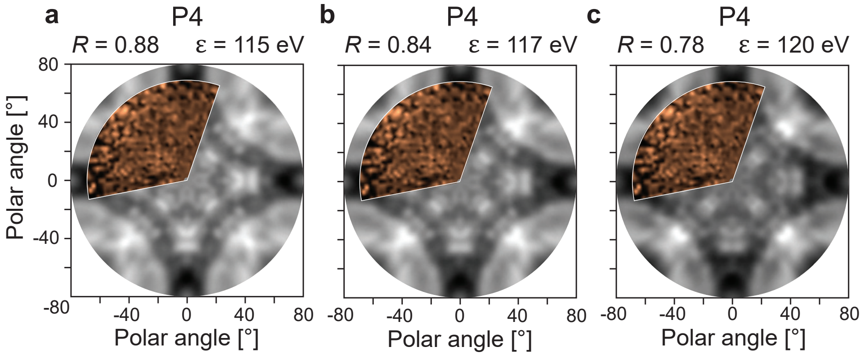

FIG. S5. The kinetic energy-dependent reliability (R) factor of component P4 achieved by comparing the measured XPD pattern to the simulated pattern from the optimized structural model at $h\nu = 250\,\text{eV}$. The R at $\varepsilon = 115\,\text{eV}$ (**a**) closest to the true kinetic energy of the signal is larger – and thus worse, than the R from several eV away from the peak position (**b**, **c**).

[1] Wilson, H. F.; Warschkow, O.; Marks, N. A.; Curson, N. J.; Schofield, S. R.; Reusch, T. C. G.; Radny, M. W.; Smith, P. V.; McKenzie, D. R.; Simmons, M. Y. Thermal Dissociation and Desorption of PH_3 on Si(001): A Reinterpretation of Spectroscopic Data. *Phys. Rev. B* **2006**, *74*, 195310.

[2] Wilson, H. F.; Warschkow, O.; Marks, N. A.; Schofield, S. R.; Curson, N. J.; Smith, P. V.; Radny, M. W.; McKenzie, D. R.; Simmons, M. Y. Phosphine Dissociation on the Si(001) Surface. *Phys. Rev. Lett.* **2004**, *93*, 226102.

[3] Warschkow, O.; Wilson, H. F.; Marks, N. A.; Schofield, S. R.; Curson, N. J.; Smith, P. V.; Radny, M. W.; McKenzie, D. R.; Simmons, M. Y. Phosphine Adsorption and Dissociation on the Si(001) Surface: An Ab Initio Survey of Structures. *Phys. Rev. B* **2005**, *72*, 125328.

[4] Wang, Y.; Bronikowski, M. J.; Hamers, R. J. An Atomically Resolved STM Study of the Interaction of Phosphine With the Silicon (001) Surface. *J. Phys. Chem.* **1994**, *98*, 5966–5973.

[5] Curson, N. J.; Schofield, S. R.; Simmons, M. Y.; Oberbeck, L.; O'Brien, J. L.; Clark, R. G. STM Characterization of the Si-P Heterodimer. *Phys. Rev. B* **2004**, *69*, 195303.

[6] McKibbin, S. R.; Clarke, W. R.; Fuhrer, A.; Reusch, T. C. G.; Simmons, M. Y. Investigating the Regrowth Surface of Si:P δ-Layers Toward Vertically Stacked Three Dimensional Devices. *Appl. Phys. Lett.* **2009**, *95*, 233111.

[7] Lin, D.-S.; Ku, T.-S.; Sheu, T.-J. Thermal Reactions of Phosphine With Si(100): A Combined Photoemission and Scanning-Tunneling-Microscopy Study. *Surf. Sci.* **1999**, *424*, 7–18.

[8] Lin, D.-S.; Miller, T.; Chiang, T.-C. Dimer Charge Asymmetry Determined by Photoemission from Epitaxial Ge on Si(100)-(2×1). *Phys. Rev. Lett.* **1991**, *67*, 2187–2190.

[9] Hollinger, G.; Himpsel, F. Probing the Transition Layer at the SiO_2-Si Interface Using Core Level Photoemission. *Appl. Phys. Lett.* **1984**, *44*, 93–95.

[10] Røst, H. I. et al. Low-Temperature Growth of Graphene on a Semiconductor. *J. Phys. Chem. C* **2021**, *125*, 4243–4252.

[11] Keizer, J. G.; Koelling, S.; Koenraad, P. M.; Simmons, M. Y. Suppressing Segregation in Highly Phosphorus Doped Silicon Monolayers. *ACS Nano* **2015**, *9*, 12537–12541.

[12] Song, F.; Monsen, Å.; Li, Z. S.; Choi, E.-M.; MacManus-Driscoll, J. L.; Xiong, J.; Jia, Q. X.; Wahlström, E.; Wells, J. W. Extracting the Near Surface Stoichiometry of $BiFe_{0.5}Mn_{0.5}O_3$ Thin Films; a Finite Element Maximum Entropy Approach. *Surf. Sci.* **2012**, *606*, 1771–1776.

[13] Oberbeck, L.; Curson, N. J.; Hallam, T.; Simmons, M. Y.; Bilger, G.; Clark, R. G. Measurement of Phosphorus Segregation in Silicon at the Atomic Scale Using Scanning Tunneling Microscopy. *Appl. Phys. Lett.* **2004**, *85*, 1359–1361.

[14] Goh, K. E. J.; Oberbeck, L.; Simmons, M. Y.; Hamilton, A. R.; Clark, R. G. Effect of Encapsulation Temperature on Si:P Delta-Doped Layers. *Appl. Phys. Lett.* **2004**, *85*, 4953–4955.

[15] Keizer, J. G.; McKibbin, S. R.; Simmons, M. Y. The Impact of Dopant Segregation on the Maximum Carrier Density in Si:P Multilayers. *ACS Nano* **2015**, *9*, 7080–7084.

[16] Kravchenko, S.; Sarachik, M. P. Metal–Insulator Transition in Two-Dimensional Electron Systems. *Rep. Prog. Phys.* **2003**, *67*, 1.

[17] McKibbin, S.; Polley, C.; Scappucci, G.; Keizer, J.; Simmons, M. Low Resistivity, Super-Saturation Phosphorus-in-Silicon Monolayer Doping. *Appl. Phys. Lett.* **2014**, *104*, 123502.

[18] Holt, A. J.; Mahatha, S. K.; Stan, R.-M.; Strand, F. S.; Nyborg, T.; Curcio, D.; Schenk, A. K.; Cooil, S. P.; Bianchi, M.; Wells, J. W.; Hofmann, P.; Miwa, J. A. Observation and Origin of the Δ Manifold in Si:P δ Layers. *Phys. Rev. B* **2020**, *101*, 121402.

[19] Seah, M. P.; Dench, W. A. Quantitative Electron Spectroscopy of Surfaces: A Standard Data Base for Electron Inelastic Mean Free Paths in Solids. *Surf. Interface Anal.* **1979**, *1*, 2–11.

[20] Ramstad, A.; Brocks, G.; Kelly, P. J. Theoretical Study of the Si(100) Surface Reconstruction. *Phys. Rev. B* **1995**, *51*, 14504–14523.

[21] García de Abajo, F. J.; Van Hove, M. A.; Fadley, C. S. Multiple Scattering of Electrons in Solids and Molecules: A Cluster-Model Approach. *Phys. Rev. B* **2001**, *63*, 075404.

[22] Tang, S.; Freeman, A. J.; Delley, B. Structure of the Si(100) 2×1 Surface: Total-Energy and Force Analysis of the Dimer Models. *Phys. Rev. B* **1992**, *45*, 1776–1783.

[23] Cataldo, L.; Choua, S.; Berclaz, T.; Geoffroy, M.; Mézailles, N.; Ricard, L.; Mathey, F.; Floch, P. L. Formation of a Phosphorus–Phosphorus Bond by Successive One-Electron Reductions of a Two–Phosphinines–Containing Macrocycle: Crystal Structures, EPR, and DFT Investigations. *J. Am. Chem. Soc.* **2001**, *123*, 6654–6661.

[24] Woodruff, D. Adsorbate Structure Determination Using Photoelectron Diffraction: Methods and Applications. *Surf. Sci. Rep.* **2007**, *62*, 1–38.
